# Engineering Gene Networks to Probe Embryonic Pattern Formation in Flies

**DOI:** 10.1371/journal.pbio.0030104

**Published:** 2005-02-22

When it comes to making the perfect body, it's all about expressing the right genes in the right place at the right time. This process begins even before the sperm and egg combine to form a zygote, because maternal factors are laid down in the egg that help establish the key axes of the body. After fertilization, precisely coordinated interactions between proteins called morphogens and a network of gene regulators establish a fly's anterior–posterior axis and its pattern of segments in just three hours.

In a new study, Mark Isalan, Caroline Lemerle, and Luis Serrano simulated segmentation patterning by creating a synthetic embryo and engineering an artificial version of the gap gene network, the first patterning genes expressed in the zygote. This simple system, combined with computer simulations to test network parameters, identifies significant features of the complex embryo and could do the same for other complex biological systems.[Fig pbio-0030104-g001]

**Figure pbio-0030104-g001:**
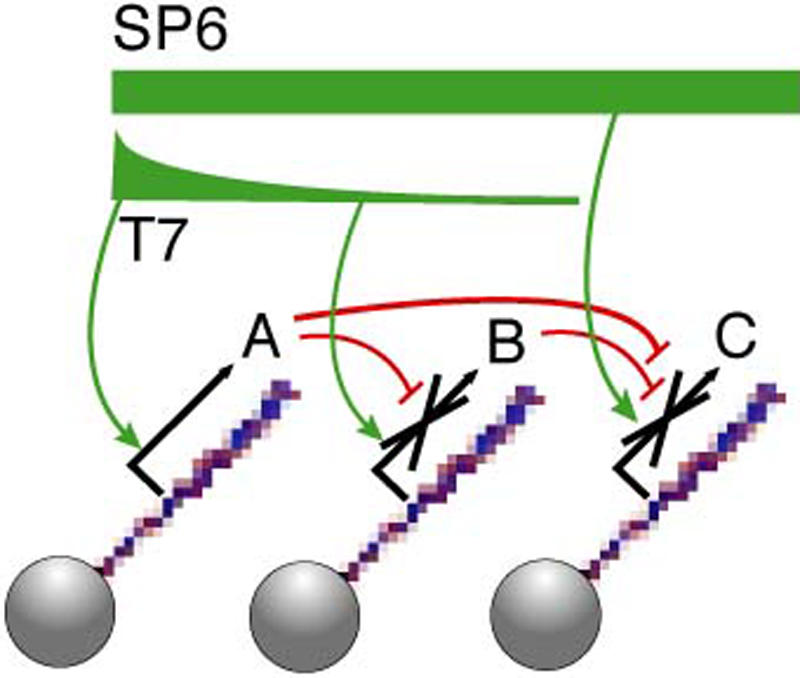
An artificial network to study patterning in development

One of the first molecules to act is the Bicoid protein. This morphogen is present in a concentration gradient—highest at the future head end. Different gap genes (so-called because their mutations create gaps in the segmentation pattern) respond to different levels of Bicoid, and are therefore switched on in different parts of the embryo. Expressed gap genes in turn modulate each other's activity. In the fruitfly, all of this action takes place while the embryo is a syncytium—having many nuclei but no cell membranes to separate them.

Isalan et al. created a model of segmentation patterning by using a tiny plastic chamber containing various purified genes, proteins, metabolites, and cell extracts to mimic the gap gene network. Some of the genes were attached to magnetic microbeads, so that their location could be controlled by magnets anchored to the bottom of the chamber.

The authors investigated a number of open questions about pattern formation, including how a morphogen diffusing from a local source generates an expression pattern along a gradient and how transcriptional repression sets pattern boundaries. After testing the system to mimic a simple network of sequential gene transcription and repression, the authors increased the components and connectivity of the network, starting with systems that had no repression interactions and moving on to systems that had different levels of cross-repression. Patterns generated by networks involving repression were much different from those generated by networks lacking repression, fitting with observations that patterning boundaries in living flies require cross-repression.

But even the unrepressed system generated reproducible patterns, possibly caused by simple competition between the proteins. While such a situation likely bears little resemblance to that inside a fly egg, the authors suggest that any such competition effects would have to be tested in flies. In any case, this simplified approach can test hypotheses of how simple networks might evolve inside a cell. And since many aspects of Drosophila embryonic patterning remain obscure, these synthetic chambers will provide a powerful resource for testing different hypotheses.

